# Supplementary material for: Crystal structure of MAGEA4 MHD-RAD18 R6BD reveals a flipped binding mode compared to AlphaFold2 prediction
Source: EMBO J. 2024 Jun 21;43(14):1. doi: 10.1038/s44318-024-00140-2 (PMC11251181; doi:10.1038/s44318-024-00140-2)
Supplement: Supplementary file 1 — Table EV1 [file 44318_2024_140_MOESM1_ESM.docx]

**Table EV1. X-ray** **data collection and refinement statistics of the MAGEA4 MHD-RAD18 R6BD complex**

|  | **MAGEA4 MHD-RAD18 R6BD complex** (PDB 9BD3) |
| --- | --- |
| **Data collection** |  |
| Space group | P 65 |
| Cell dimensions |  |
| *a*, *b*, *c* (Å) | 85.21, 85.21, 206.08 |
| α, β, γ (°) | 90.00, 90.00, 120.00 |
| Resolution (Å) | 42.24 – 2.58 (2.67 – 2.58) * |
| *R*_merge_ | 0.0654 (0.8175) |
| *R*_pim_ | 0.0284 (0.3607) |
| *CC_1/2_* | 0.999 (0.877) |
| *I* / σ*I* | 19.04 (3.79) |
| Completeness (%) | 98.23 (98.60) |
| Redundancy | 6.1 (6.3) |
|  |  |
| **Refinement** |  |
| Resolution (Å) | 2.58 |
| No. reflections | 26,085 (2,599) |
| *R*_work_ / *R*_free_ | 0.1912 / 0.2262 |
| No. atoms | 3,756 |
| Protein | 3,674 |
| Ligand/ion | 0 |
| Water | 82 |
| *B*-factors | 61.65 |
| Protein | 61.79 |
| Ligand/ion | N/A |
| Water | 55.11 |
| R.m.s. deviations |  |
| Bond lengths (Å) | 0.003 |
| Bond angles (°) | 0.57 |
| Ramachandran |  |
| favored (%) | 96.90 |
| allowed (%) | 3.10 |
| outliers (%) | 0.0 |

*Values in parentheses are for highest-resolution shell.
