# Supplementary material for: Crystal structure of MAGEA4 MHD-RAD18 R6BD reveals a flipped binding mode compared to AlphaFold2 prediction
Source: EMBO J. 2024 Jun 21;43(14):1. doi: 10.1038/s44318-024-00140-2 (PMC11251181; doi:10.1038/s44318-024-00140-2)
Supplement: Supplementary file 3 — Expanded View Figures [file 44318_2024_140_MOESM3_ESM.pdf]

Expanded View Figures

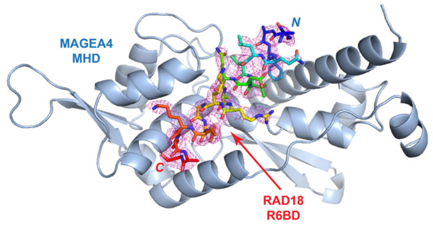

Figure EV1. Omit map of the RAD18 R6BD in the MAGEA4 MHD-bound complex.

Each unit cell contains two protomers with similar conformations. For clarity, only protomer 1 (chain A and chain B) is shown here. The MAGEA4 MHD (chain A) is shown in the cartoon representation and colored in light blue. The RAD18 R6BD (chain B) is shown in the stick model and color in rainbow with the N-terminus in blue and C-terminus in red. The purple mesh represents the 2mFo-DFc omit map of the RAD18 R6BD (chain B) contoured at 1.0 σ. The N-terminus and C-terminus of the RAD18 R6BD are labeled.

 © The Author(s)

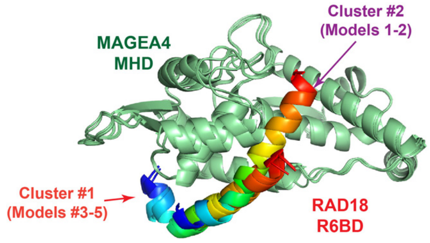

Figure EV2. Five models of the MAGE MHD-RAD18 R6BD complex.

The sequences of human MAGEA4 MHD (D101-V317) and RAD18 R6BD (H339-G366) were used as the input for the ColabFold implementation of the AlphaFold-Multimer. The MAGEA4 MHD is colored in pale green and RAD18 R6BD colored in rainbow, with N-terminus in blue and C-terminus in red. The five models of the RAD16 R6BD share similar orientations but are congregated into two clusters, with models #3-#5 into one cluster (cluster #1) that resembles the AlphaFold2 model reported by (Griffith-Jones et al, 2024) and models #1 and #2 into the other cluster (cluster #2).
